# Evidence for consistent individual differences in rat sensitivity to carbon dioxide

**DOI:** 10.1371/journal.pone.0215808

**Published:** 2019-04-24

**Authors:** Lucía Améndola, Daniel M. Weary

**Affiliations:** Animal Welfare Program, University of British Columbia, Vancouver, British Columbia, Canada; Humboldt-Universitat zu Berlin, GERMANY

## Abstract

Carbon dioxide (CO_2_) gradual-fill is commonly used to kill laboratory rats, but this use remains controversial due to a lack of agreement between studies. Inconsistencies may arise from differences in behaviors measured (e.g. active versus passive behaviors), in how rats cope with threats, or in rat sensitivity to CO_2_. The aims of the current study were to 1) describe active and passive responses during CO_2_ forced exposure, 2) determine if these responses are consistent within individuals and across aversive stimuli, 3) assess individual differences in aversion to CO_2_ in aversion-avoidance and approach-avoidance tests and 4) determine how responses in aversion tests relate to individual differences in behavior during forced exposure. Twelve Sprague Dawley female rats were exposed twice to three treatments: CO_2_, oxygen (O_2_), and fox scent, and were exposed to CO_2_ twice in each aversion test. The change in behavior from baseline was higher for rearing and locomotion when rats were exposed to CO_2_ than when exposed to O_2_ and fox scent. Responses varied among rats but were consistent across multiple tests within rats. For example, rearing was consistent within individuals between two exposures to CO_2_. Similarly, the strength of aversion was consistent within individuals across multiple exposures to CO_2_ in aversion-avoidance and approach-avoidance testing. Latency to avoid CO_2_ in aversion-avoidance tests was negatively correlated with rearing during CO_2_ forced exposure. Collectively, these results indicate that rat responses to CO_2_ vary between (but are consistent within) individuals, suggesting that rats vary in CO_2_ sensitivity. However, even the less responsive rats avoided CO_2_ concentrations far below those necessary to achieve unconsciousness, indicating that all rats likely experience negative states when euthanized with CO_2_.

## Introduction

Carbon dioxide (CO_2_) is a widely used but controversial method of killing laboratory rodents [1]. Guidelines and regulations commonly accept this agent as a ‘humane’ killing method (e.g. [2–4]), implying that animals should not experience high arousal negative emotions during exposure, including pain, fear, distress, or anxiety.

Here we refer to emotional responses as objectively observable behavioural, physiological and brain responses to stimuli [5]. Emotions in animals are often inferred from behavioral responses during forced exposure to a noxious agent. The frequency, duration and intensity of rat active defense responses (e.g. increased locomotion, rearing, and the attempts to escape the cage, etc.) have been interpreted as signs of a negative emotional experience during CO_2_ exposure (e.g. [6–9]). Choice and between-motivation tests, which are based on the animal’s motivation to approach desired and avoid undesired states (see [10]), have also been used to assess rat emotions elicited by CO_2_. Choice tests provide rats with two mutually exclusive conditions (e.g. a chamber pre-filled with high CO_2_% and low CO_2_% pre-filled chamber), the amount of time animals spend in each condition is indicative of preference (e.g. [11]). Between-motivation tests compare aversion to CO_2_ with motivation to approach or avoid a stimulus thought to elicit positive or negative emotions, respectively. For example, in aversion-avoidance tests the cost of avoiding CO_2_ is exposure to an aversive brightly light chamber (e.g. [12]), and in approach-avoidance tests the cost of avoiding CO_2_ is loss of a sweet food reward (e.g. [13]).

Choice tests have shown that rats prefer (total time in the chamber between 36 and 51 s) to be exposed to <1% CO_2_ than to be exposed to 25.5% CO_2_ (total time in the chamber around 2.1 s) or 50.8% CO_2_ (total time in the chamber around 0.7 s) [11–14]. Research using between-motivation tests has consistently shown that rats find CO_2_ aversive and that they are motivated to avoid CO_2_ concentrations between 14% and 18% (e.g. [13,15–17]), well below the concentrations required to render animals recumbent (approximately 33% CO_2_) [9]. The results of forced exposure tests have been less consistent. Some studies have found behavioral responses in rats exposed to CO_2_ (e.g. [8,9,16,18]), but others have reported little or no response (e.g. [6,19,20]). Results from choice and between-motivation tests indicate that CO_2_ elicits negative states which rats are motivated to avoid, indicating that CO_2_ is not a humane killing method for rats, but the lack of agreement between studies using forced exposure tests may help perpetuate the use of this method. Indeed, this lack of consistency is cited in recent reviews supporting the use of CO_2_ as a humane killing method [21,22].

Research examining CO_2_ as a euthanasia agent has only considered active defense responses to forced exposure, but rats also show passive responses (freezing/immobility) [23,24], and these responses have been the focus of research on the use of CO_2_ as an anxiogenic (e.g. [25–27]). As some of the previous euthanasia research may have failed to find effects because only active responses were considered, the first aim of our study was to examine both passive and active defense responses during CO_2_ gradual-fill forced exposure. We predicted that when exposed to CO_2_, rat passive and active defense responses would increase from baseline more than when exposed to Oxygen (O_2_) as a control.

A number of studies have reported between-rat variation in response to gradual-fill CO_2_. For example, previous studies from our research group found that the frequency of escape behaviors ranged between individuals from zero to 34 [9], and that about 50% of the rats tested showed increased locomotion [17]. Smith and Harrap [20] found that about 20% of rats climbed or moved around the perimeter of the cage in response to CO_2_ exposure. Leach and colleagues [11,14], using choice tests, reported high inter-individual variability in responses. Aversion to CO_2_ is also variable among rats. For example, one study using aversion-avoidance testing found that the time to avoid CO_2_ varied among rats from 7 to 48 s [12], and a study using approach-avoidance found that the concentration of CO_2_ avoided varied from 5% to 25% [17].

Evidence of variability in rat responses to CO_2_ in motivational tests suggests variation in CO_2_ sensitivity. It has been well documented that humans vary in their emotional responses to CO_2_. For example, following a double inhalation between 9 and 35% CO_2_, approximately 50% of healthy humans experience anxiety [28], and a single inhalation of 35% CO_2_ elicits panic in between 43 and 94% of patients with panic disorder (PD) (for a review, see [29]). Heightened sensitivity to CO_2_ in humans may be associated with a false suffocation alarm (i.e. an inappropriate activation of systems that monitor suffocation) [30].

Personality differences–extensively documented in different animal taxa [31]–may account for variation in rat responses to CO_2_ in a situation-dependent manner. We define personality following Réale et al. [32] as individual differences consistent across time and contexts. Variation in rat responses to CO_2_ may reflect different behavioral strategies. For example, de Boer and Koolhaas [24] found that some rats attempted to bury a prod that delivered shocks, but others moved away from the prod and remained immobile. If consistent within rat, this variation between rats could be related to more general personality differences in how individuals respond to threatening stimuli. The second aim of our study was to determine if rats consistently vary in behavioral strategies when exposed to CO_2_. If variation in response to CO_2_ is reflective of individual differences in response to threatening stimuli in general, we expected that responses to CO_2_ would be related to those to fox scent, and that passive and active defense responses would be consistent within and between stimuli.

Human variation in CO_2_ sensitivity is consistent between repeated exposures (e.g. [33–35]). In rats, individual differences in sensitivity could be reflected in consistent behavioural responses across time, regardless of the type of defence behaviour expressed (i.e. active and passive responses), and situational (e.g. forced exposure and aversion tests). Thus, the third aim of our study was to assess rat variation in CO_2_ sensitivity. Our hypothesis was that variation in rat responses to CO_2_ is reflective of CO_2_ sensitivity, and we predicted that rat responses to CO_2_ would be consistent within and between aversion-avoidance, approach-avoidance, and forced exposure tests.

## Methodology

All procedures were approved by The University of British Columbia Animal Care Committee (protocol number: A15-0071) and were performed in accordance with the guidelines on care and use of rodents in research, established by the Canadian Council on Animal Care.

### Subjects and housing

Previous work by our group has shown that a sample size of approximately 8 rats is necessary to detect treatment differences (e.g. [9,13,16,17]), but measures of individual differences typically require larger sample sizes. Therefore, we used twelve female Sprague-Dawley rats, all obtained as surplus stock from the University of British Columbia. Rats were not part of any experimental procedure prior to this study. One animal showed signs of ill health, was treated with an anti-inflammatory and was not used in the tests. Rats were individually marked with a permanent marker (Ketchum Manufacturing Inc., ON, Canada), and housed in groups of three in two polycarbonate cages (Lab Products, Inc. DE, USA) connected by a red tinted polycarbonate tube (7.6 cm diameter, 15 cm long), to provide rats with more home-cage space. One cage was smaller (20 x 45 x 24 cm) and contained food (Rat Diet PMI 5012, Lab Diets, Land O'Lakes, Inc., MN, USA), tap water and bedding material (1/4 inch PBP with Enrichment Bedding, Biofresh, Absorption Corp, WA, USA), while the other cage was bigger (20 x 50 x 40 cm) and contained bedding material (1/4 inch PBP with Enrichment Bedding, Biofresh, Absorption Corp, WA, USA), a PVC tube, and a cardboard box. All cages and bedding were replaced once a week on Thursday after 1700 h, to reduce the risk that any effects from cage-changing (which can last several hours [36]) affected our results. All animals had ad libitum access to food (Rat Diet PMI 5012, Lab Diets, Land O'Lakes, Inc., MN, USA) and tap water and received daily treats (oats and shredded coconut). Rats were kept under reverse lighting (dark period from 0800 h to 2000 h). Temperature and humidity were controlled and averaged (mean ± standard deviation) 24 ± 0.6°C and 52 ± 5.8%, respectively. Rats were 9 months old and weighed 403 ± 54 g at the end of the study.

### Handling and experimental room

All rats were habituated to handling during a 10-day period before experiments started. In all experiments, each rat was tested only once per day. Tests were performed between 0900 h and 1700 h, and each rat was tested at similar times within and across all experiments. All tests were performed in an experimental room with a ventilation rate of 12 room air changes per h with a wireless controlled lighting system programmed to deliver light at 615 nm (red light; Philips HUE Personal Wireless Lighting BR30 LED, Koninklijke Philips, AMS, Netherlands). The oxygen analyzer was kept on during all habituation and testing sessions. For all experiments, habituation, and training, rats were individually transported into the experimental room in a transport cage covered with black plastic. Once in the experimental room, rats were left in the transport cage undisturbed for 5 min. Subjects were isolated from cage-mates for a maximum of 40 min per day.

### Experiment 1: Forced exposure

#### Apparatus

**Fig 1:**
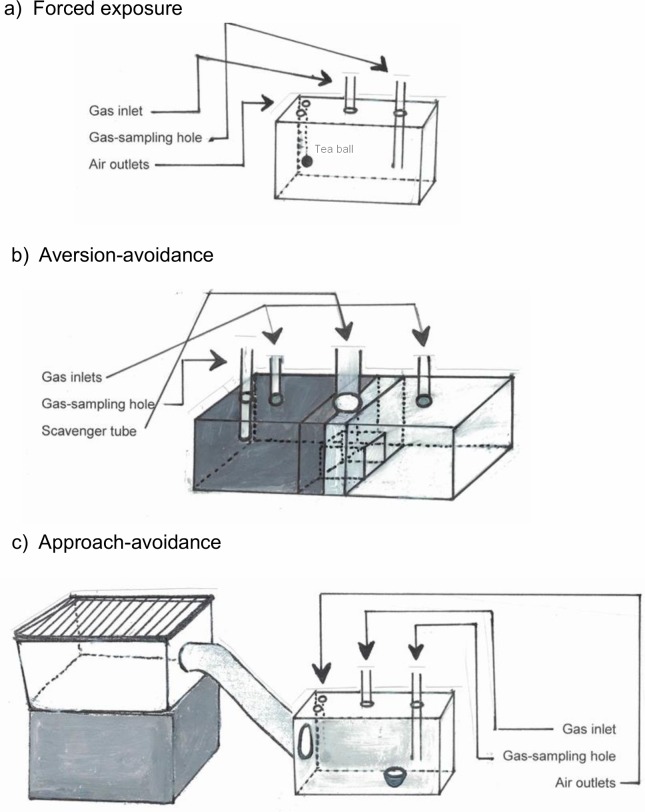
Experimental apparatus. Apparatus used in the a) forced exposure, b) aversion-avoidance, and c) approach-avoidance experiments (see supporting information: S2 Appendix).

Forced exposure tests were performed in plastic cages (20 x 45 x 24 cm) with bedding (1/4 inch PBP with Enrichment Bedding, Biofresh, Absorption Corp, WA, USA), covered with an acrylic glass lid that contained a gas inlet, a gas-sampling hole, two air outlets (covered with a mesh), and a metallic tea bag attached between the air outlets (Fig 1A).

CO_2_ was delivered from compressed gas cylinders (Praxair, BC, Canada), through a clear vinyl tube inserted in the gas inlets. Gas flow was regulated using a flow meter (CO_2_: Western Medica, OH, USA). A wall-mounted outlet (Amico Corporation, ON, Canada) delivered O_2_ through a clear vinyl tube inserted in the gas inlets; flow was regulated with a flow meter integrated in an anesthetic machine (VetEquip, Inc., CA, USA), with no anesthetic used. The sampling tube was attached to an oxygen analyzer (Series 200, Alpha Omega Instrument Corporation, RI, USA).

#### Experimental design

Twelve rats were exposed during four consecutive days, once to each of three treatments: CO_2_ gradual-fill (18.5% chamber vol. min^-1^), oxygen (O_2_; 3.5 L min^-1^) gradual-fill (as a control), and fox scent (as a passive response eliciting stimulus; TMT at 5 μl at 3.87 μmol, Scotts Miracle-Gro Company, OH, USA) [37]. As a part of another study, rats were also exposed to a bleach treatment (2 ml; The Clorox Company, CA, USA; results are not reported further but experimental procedures and data are provided as supporting information: S1 Appendix and S3 Dataset). Order of exposure was allocated using three 4x4 Latin squares (four rats and four treatments: CO_2_, O_2_, fox scent and bleach). Three days later, the same twelve rats were re-exposed to the treatments, allocating treatment order in three different 4x4 Latin squares (a timeline of the experiments is presented as supporting information: S2 Appendix).

#### Testing procedure

Rats were individually placed in the experimental cage covered with the baseline lid, and remained there for 5 min. The lid was then replaced with the experimental lid. For the fox scent treatment, the tea ball containing filter paper with 5 μl of fox scent was attached to the experimental lid. For CO_2_ and O_2_ treatments, the tea ball attached to the experimental lid was empty. After the experimental lid was in place the gas flow started. Tests were stopped when CO_2_ reached 25% in the experimental cage, after 120 s in O_2_ tests and after 15 min in the fox scent treatment. After this last treatment no tests were performed in the room for at least 20 min to allow the ventilation system to make a minimum of four complete room air changes.

After each test, the experimental cage and lids were cleaned with Quatricide (Pharmacal Research Laboratories, Naugatuck, CT, USA), rinsed with water, cleaned with ethanol, and bedding was replaced. All forced exposure tests were performed under red light.

#### Behavioral observations

All forced exposure tests were video recorded. The videos were divided into baseline (60 s before any test) and initial response periods (first 60 s of the test). In the fox scent treatment during re-exposure, two animals were excluded because of lost video. For all treatments, videos were scored using Solomon (Solomon coder Version beta 15.11.19). A trained observer, blind to rat identity and treatment, recorded active and passive behavioral responses (Table 1). To estimate inter-observer reliability, another independent observer, again blind to treatment scored 20 of the videos. Inter-observer reliability was assessed using Pearson correlation tests following Martin and Bateson [38] (rearing: r = 0.91, line-crossing: r = 0.77, immobility time: r = 0.99, bedding manipulation: r = 0.76; lid-pushing was too rare to assess).

**Table 1:** Description of active and passive behavioral responses of rats during forced exposure.

Type of response	Behavior	Description
Active	Rearing	Raising the upper body on the hind limbs, in a vertical position with both front paws off the ground (frequency)
	Line-crossing	Horizontal locomotor activity that results in the rat’s forepaws crossing a line that divides the length of the chamber in half (frequency)
	Lid-pushing	Push at the cage lid with the nose or front paws (frequency)
	Bedding manipulation	Displacement (pushing, shoveling, flicking, or digging) of bedding material with front and/or back paws (frequency)
Passive	Immobility time	Absence of movement, except for small and slow lateral movements of the head between frames. Behavior measured as time(s) spent immobile

### Experiment 2: Aversion-avoidance

#### Apparatus

The aversion-avoidance apparatus consisted of an acrylic glass light-dark box consisting of two compartments (14 x 27 x 30 cm each), connected by a smaller buffer compartment (10 x 14 x 30 cm). The light compartment was covered with white plastic, and illuminated by two bulbs placed above the lid. The bulbs provided a light intensity of 1650 lux, measured at the bottom of the compartment. The dark compartment was covered with opaque black plastic. All compartments contained bedding (1/4 inch PBP with Enrichment Bedding, Biofresh, Absorption Corp, WA, USA). Doorways of the buffer compartment were covered with plastic flaps. The light-dark box was covered with an acrylic glass lid. The lid contained a gas inlet in the middle of each compartment, a gas-sampling hole, and a scavenger tube attached to a hole in the middle of the buffer compartment. The portion of the lid corresponding to the dark compartment was covered with opaque black plastic (Fig 1B).

Air was regulated using a flow meter (Dwyer instruments, Inc., NI, USA), and delivered from a compressed gas cylinder through a clear vinyl tube inserted in the gas inlets. CO_2_ was regulated and delivered as described for Experiment 1.

#### Habituation and training

Rats were habituated to the light-dark box over four consecutive days. Each subject was placed in the light compartment of the apparatus and left to explore for 30 min. On Day 1, rats were placed in the apparatus under red light. From Day 2 onwards, the light level was 1650 lux in the light compartment. On the third and fourth day, airflow (3.5 L min^-1^) was delivered in both compartments.

#### Experimental design

The same rats tested in Experiment 1 were use in this experiment. Rats were exposed twice to CO_2_ (19% chamber vol. min^-1^) during two consecutive days (see supporting information: S2 Appendix).

#### Testing procedure

Rats were individually placed in the bright compartment of the dark-light box and left for 30 min to explore the apparatus with airflow delivered to both compartments. All subjects settled down in the dark compartment for at least 10 min by the end of the 30-min period. CO_2_ flow was then started in the dark compartment. The test stopped when the rat moved from the dark to the light compartment (i.e. shoulders crossed from the buffer compartment to the light compartment); the latency to leave the dark chamber was recorded as the dependent variable. The dark-light box was cleaned with Quatricide, rinsed with water, and the bedding replaced after each test.

### Experiment 3: Approach-avoidance

#### Apparatus

The approach-avoidance apparatus consisted of each rat’s bigger home cage placed 20 cm higher (top cage) than a smaller bottom cage (20 x 45 x 24 cm). A transparent acrylic glass tube (10 cm diameter, and 45 cm length), with cleats to prevent slipping, connected the two cages. An acrylic glass sliding door (10 x 10 cm) was attached between the connection tube and the top cage. Both cages contained bedding (1/4 inch PBP with Enrichment Bedding, Biofresh, Absorption Corp, WA, USA). The bottom cage was covered with an acrylic glass lid that contained two air outlets, a gas inlet, and a gas sampling tube in the middle of the cage (Fig 1C). CO_2_ and O_2_ were delivered and regulated following Experiment 1.

#### Habituation and training

Rats were trained for approach-avoidance testing for 12 days. Each rat was placed in the top cage of the apparatus and was able to move freely throughout for 5 min. After this period, if the rat was in the bottom cage, it was encouraged to return to the top cage with a reward (one Cheerio; Honey Nut Cheerios TM, General Mills Inc., MN, USA). The rat was kept in the top cage for 2 min by closing the sliding door, and 20 Cheerios were placed in the bottom cage. The sliding door was then opened and the rat was allowed to descend to the bottom cage and eat the Cheerios; as soon as the rat returned to the top cage the sliding door was again closed. O_2_ (3.5 L min^-1^) was introduced into the bottom cage as soon as the rat started eating.

#### Experimental design

The same rats were tested as those used in Experiments 1 and 2. Rats were exposed twice to CO_2_ (18.5% chamber vol. min^-1^).

#### Testing procedure

Rats were introduced into the top cage of the approach-avoidance apparatus and allowed to explore the apparatus for 5 min. Rats were then encouraged to return to the top cage (if not already there) using a Cheerio as a treat, and the door was closed. After 2 min, twenty Cheerios were placed in the bottom cage, and the rat was allowed to descend. Gradual-fill of CO_2_ began as soon as the rats started eating the Cheerios. The test stopped once the rat left the bottom cage (i.e. shoulders crossed into the connecting tube); latency to leave the bottom chamber was recorded as the dependent variable. After each test, the bottom cage was cleaned with Quatricide, rinsed with water, and the bedding was replaced.

### Assessment of CO_2_ concentrations

To describe the changes in CO_2_ concentration during the gradual-fill procedure, nine trials were conducted in both the aversion- and approach-avoidance cages with no animals present. CO_2_ was introduced into the aversion-avoidance apparatus at a flow rate of 19% chamber vol. min^-1^. In the approach-avoidance apparatus CO_2_ was introduced at 18.5% chamber vol. min^-1^. The oxygen analyzer, attached to the gas sampling tube (Fig 1B and 1C), was video recorded during the filling process (5 min). Changes in O_2_ were used to estimate CO_2_ concentration at each time point using the formula CO_2 (t = x)_ = 100 –([O_2 (t = x)_ * 100] / O_2 (t = 0)_.

### Data analysis

All analyses were conducted with R (R Development Core Team, Version 3.4.1) and RStudio (RStudio, Inc., Version 1.0.136). Results are reported as means ± standard errors.

#### Experiment 1: Forced exposure

To compare rat responses between the three different treatments (i.e. CO_2_, O_2_ and fox scent), we used Linear Mixed Models. The response variables were rearing, line-crossing and immobility time, all expressed as change from baseline. In the models we included treatment, exposure number (exposure and re-exposure) and previous exposure to bleach (the day before the test; 0 = yes, 1 = no) as fixed factors, time of the day (h) as a covariate, and the interaction between treatment and exposure number, previous exposure to bleach and time of the day. We also included rat identity nested within cage as random intercept. The significance of the random intercept was assessed though the likelihood ratio test (LRT). Tukey *post hoc* tests were used to explore significant effects. Normality of the residuals was visually assessed.

To assess consistency of rat responses between exposures within treatment, we used Pearson correlation (CO_2_: rearing and line-crossing; fox scent: immobility time) or Kendall rank correlation with normal approximation and continuity correction for ties if responses were not normally distributed (CO_2_: immobility time; fox scent: rearing and line-crossing). Consistency between treatments is not reported due to low consistency within fox scent treatment.

#### Experiments 2 and 3: Aversion- and approach-avoidance

To explore variability in the strength of aversion to CO_2_ within each aversion test, two Linear Mixed Models were used with the response variable latency to avoid CO_2_. The models included exposure (exposure vs. re-exposure) as a fixed factor, time of the day as a covariate, and rat identity nested within cage as random intercept. We evaluated the significance of the random intercept though LRT. Normality of the residuals was visually assessed.

Consistency within aversion- and approach-avoidance tests was assessed using Pearson correlation. The average latency to avoid CO_2_ per rat in each test was used to analyze the relationship between aversion- and approach-avoidance tests using Pearson correlation. Within rat, the average rearing during CO_2_ forced exposure (for exposure and re-exposure; as these were found to be consistent) was compared with the average latency to avoid CO_2_ in aversion- and approach-avoidance tests (for exposure and re-exposure; again consistent), using Pearson correlation.

## Results

### Active and passive responses during forced exposure

Lid-pushing was rare; one rat pushed four times during the first exposure to CO_2_. Bedding manipulation was observed in one trial during baseline testing, and in 6 trials during the first exposure (2 rats for CO_2_, 3 rats for O_2_, and 1 rat for fox scent); the frequency of manipulation within test ranged between 1 and 6. These variables were not further analyzed.

**Fig 2:**
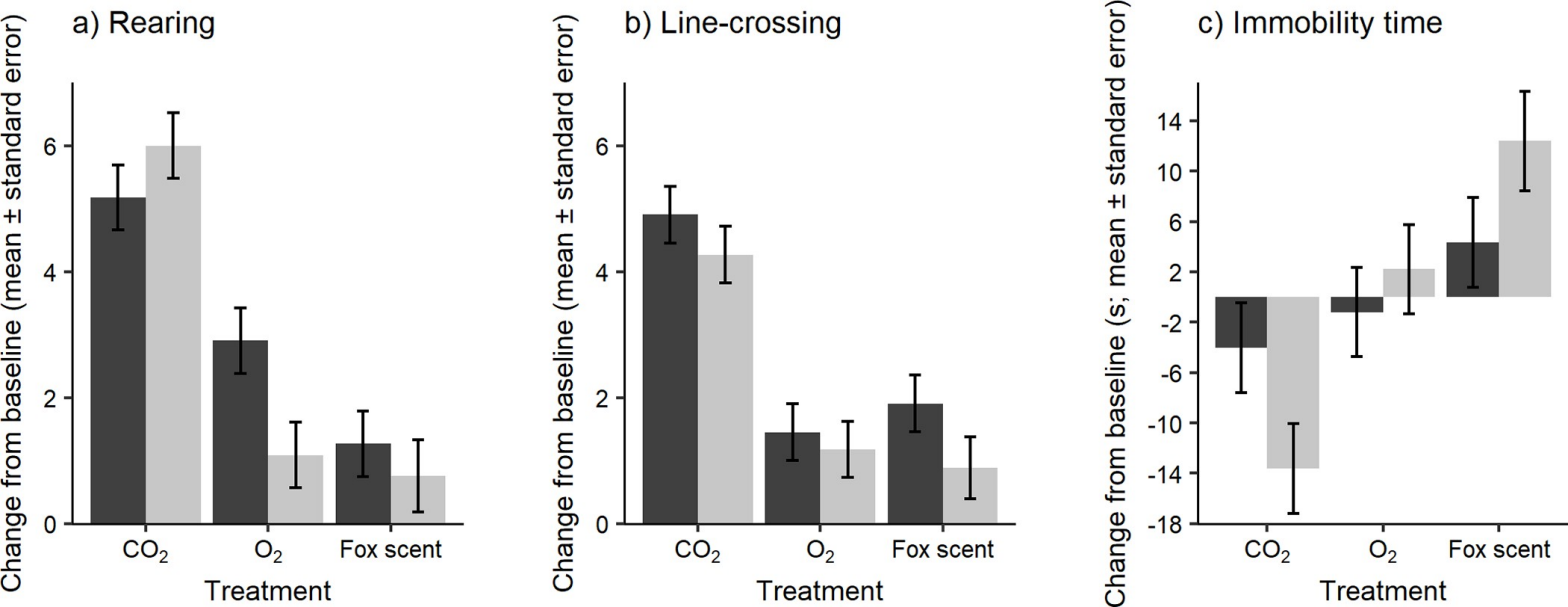
Responses to forced exposure. Rat behavior during exposure (dark bar) and re-exposure (light bar); n = 11 rats for all conditions except for n = 9 rats fox scent re- exposure.

We found a tendency for an interaction between treatment (i.e. CO_2_, O_2_, and fox scent) and exposure number (i.e. exposure and re-exposure) for rearing behavior (F = 3.05, df = 2, 42, p = 0.06). *Post hoc* analysis showed that the change in rearing (from baseline) tended to be greater during exposure and was significantly greater during re-exposure with CO_2_ than with O_2_ (exposure: p = 0.07; re-exposure: p < 0.001). Rearing behavior was greater during CO_2_ exposure and re-exposure than during fox treatment (exposure: p < 0.01; re-exposure: p < 0.0001). No differences were detected between O_2_ and fox scent treatments for the change in rearing from baseline during exposure and re-exposure (Fig 2A). We found no effects of time of the day (F = 0.61, df = 1,42, p = 0.44) and previous exposure to bleach (F = 0.66, df = 1,42, p = 0.42), and no evidence for an interaction between treatment and these variables (time of the day: F = 0.25, df = 2,42, p = 0.78; previous exposure to bleach: F = 0.09, df = 2,42, p = 0.92). Cage and rat identity nested in cage accounted for little of the variation in this behavior (cage: ~0% of the variation; Likelihood Ratio Test: LR < 0.0001, p ~ 1; rat identity nested in cage: 5.5% of the variation, LR = 0.36, p = 0.55).

The effect of treatment was significant for line-crossing behavior (F = 30.11, df = 2, 42, p < 0.001), with no effect of exposure number (F = 2.41, df = 1,42, p = 0.13), time of the day (F = 0.51, df = 1,42, p = 0.48) or previous exposure to bleach (F = 0.54, df = 1,42, p = 0.47). There was no interaction between treatment and exposure number (F = 0.35, df = 2,42, p = 0.70), time of the day (F = 0.37, df = 2,42, p = 0.69), or previous exposure to bleach (F = 1.18, df = 2,42, p = 0.32). The change in line-crossing (from baseline) was greater for CO_2_ than during O_2_ or fox scent (p < 0.0001 and p < 0.0001, respectively), with no difference between O_2_ and fox scent treatments (Fig 2B). The random intercept accounted for little variation in line-crossing (cage number: 2.8% of the variation, LR = 0.23, p = 0.63; rat identity nested in cage: ~0% of the variation, LR < 0.0001, p ~ 1).

We found a significant interaction between treatment and exposure number (F = 4.11, df = 2,42, p < 0.05). *Post hoc* analysis showed that the change in immobility was no different between treatments during exposure (Fig 2C). During re-exposure, rats showed less increase in immobility during CO_2_ than during O_2_ and fox scent treatments (p < 0.01 and p < 0.001, respectively). We found a significant interaction of treatment and time of the day on immobility (F = 4.93, df = 2,42, p < 0.05). For CO_2_ and O_2_ treatments, change in immobility as a function of time of the day was not significant (CO_2_: β = 1.53, t = 1.05, df = 42, p = 0.30; O_2_: β = -2.60, t = -1.86, df = 42, p = 0.07); during fox scent treatment, immobility time decreased with time of the day (β = -4.16, t = -2.56, p < 0.05). The effect of previous bleach exposure was not significant (F = 0.21, df = 1,42, p = 0.65) and we found no interaction between treatment and previous bleach exposure (F = 0.17, df = 2,42, p = 0.84). The random intercept accounted for little variation in this response (cage number: 12% of the variation; Likelihood Ratio Test: LR = 2.9, p = 0.15; rat identity nested in cage: ~0% of the variation, LR < 0.0001, p ~ 1).

### Within- and between-treatment consistency in active and passive responses

Rats were individually consistent in their rearing responses across two exposures to CO_2_ (Pearson correlation test: r = 0.62, df = 9, p < 0.05), but line-crossing and immobility time were not consistent (line-crossing: r = -0.13, df = 9, p = 0.71; immobility time Kendall rank test: tau = -0.17, p = 0.58). We found little evidence of consistency for rearing, line-crossing and immobility time within fox scent treatment (rearing: tau = 0.10, p = 0.82; line-crossing: tau = -0.37, p = 0.25; immobility time: r = 0.52, df = 7, p = 0.15).

### Consistency in the strength of aversion to CO_2_

During the last O_2_ training trial in the approach-avoidance task, rats left the cage after 237 ± 27 s. All rats avoided CO_2_ before any signs of ataxia in the aversion- and approach-avoidance tests.

During the first exposure in the aversion-avoidance test, latency to avoid CO_2_ ranged between 17 and 60 s (35 ± 4 s), which corresponds to approximately 8 and 22% CO_2_ (15 ± 1% CO_2_). During re-exposure, latency to avoid CO_2_ ranged between 11 and 70 s (33 ± 6 s), corresponding to approximately 5 and 25% CO_2_ (14 ± 2% CO_2_). Exposure and time of the day had no effect on the latency to avoid CO_2_ in the aversion-avoidance test (exposure: F = 0.62, df = 1, 9, p = 0.45; time of the day: F = 1.24, df = 1,9, p = 0.29).

For the approach-avoidance test, latency to avoid CO_2_ ranged between 11 and 54 s (23 ± 4) during the first exposure and between 9 and 47 s (28 ± 4 s) during the second exposure. These latencies correspond to approximately 4 and 19% CO_2_ (9 ± 2% CO_2_) during the first exposure, and 3 and 17% CO_2_ (11 ± 1% CO_2_) during re-exposure. No effect of repeated exposure or time of the day was detected on the latency to avoid CO_2_ (exposure: F = 2.52, df = 1,9, p = 0.15; time of the day: F = 0.14, df = 1,9, p = 0.72).

**Fig 3:**
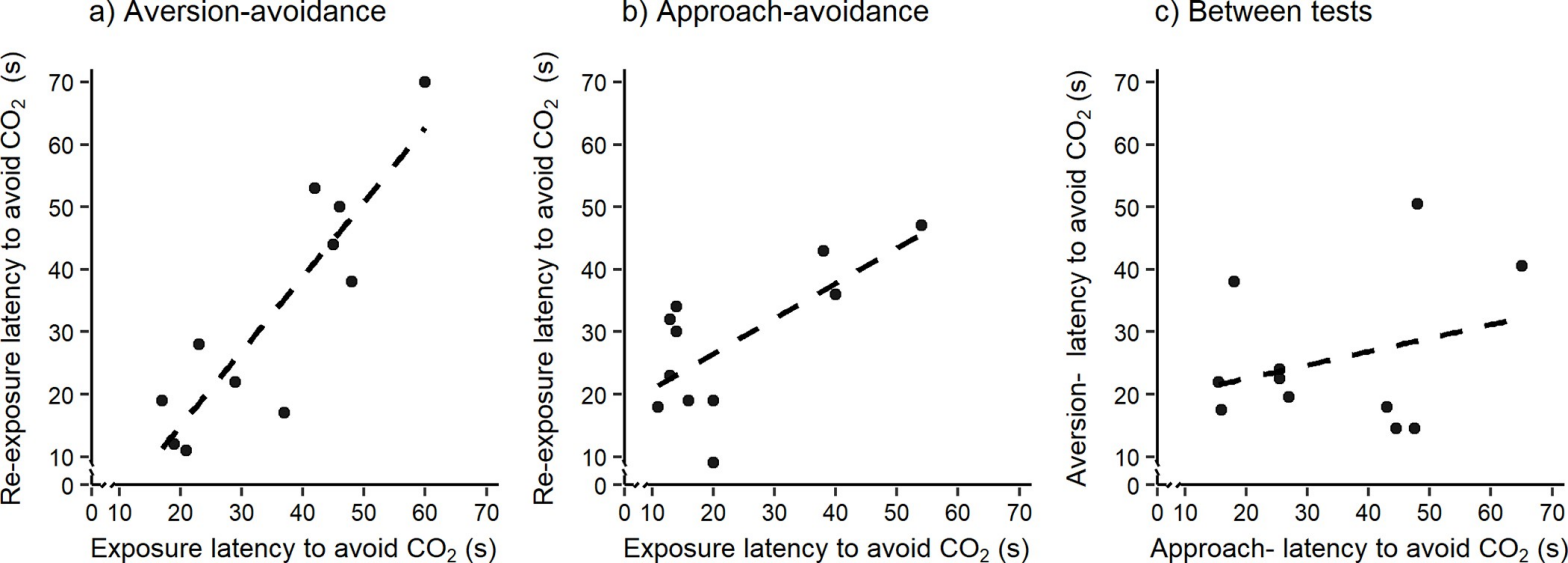
Within aversion tests consistency. Within-tests consistency between exposure and re-exposure on the latency to avoid CO_2_ in a) aversion-avoidance (n = 11 rats); b) approach-avoidance (n = 11 rats) and c) average latency to avoid CO_2_ between aversion- and approach-avoidance tests (n = 11 rats).

Cage was accounted for little variation in the latency to avoid CO_2_ in aversion- (15%) and approach-avoidance (~0%) tests (aversion-avoidance: LR = 0.11, p = 0.74; approach-avoidance: LR < 0.0001, p ~ 1). Rat identity nested within cage explained 73% (LR = 13.52, p < 0.001) and 66% (LR = 6.22, p < 0.05) of the variation in the latency to avoid CO_2_ in the aversion- and approach-avoidance tests, respectively. Within aversion tests, the latency to avoid CO_2_ was consistent (aversion-avoidance: r = 0.88, df = 9, p < 0.001; approach-avoidance: r = 0.69, df = 9, p = 0.02; Fig 3A and 3B). However, aversion to CO_2_ was not correlated between aversion- and approach-avoidance tests (r = 0.29, df = 9, p = 0.38; Fig 3C).

### Responses to forced exposure and strength of aversion to CO_2_

**Fig 4:**
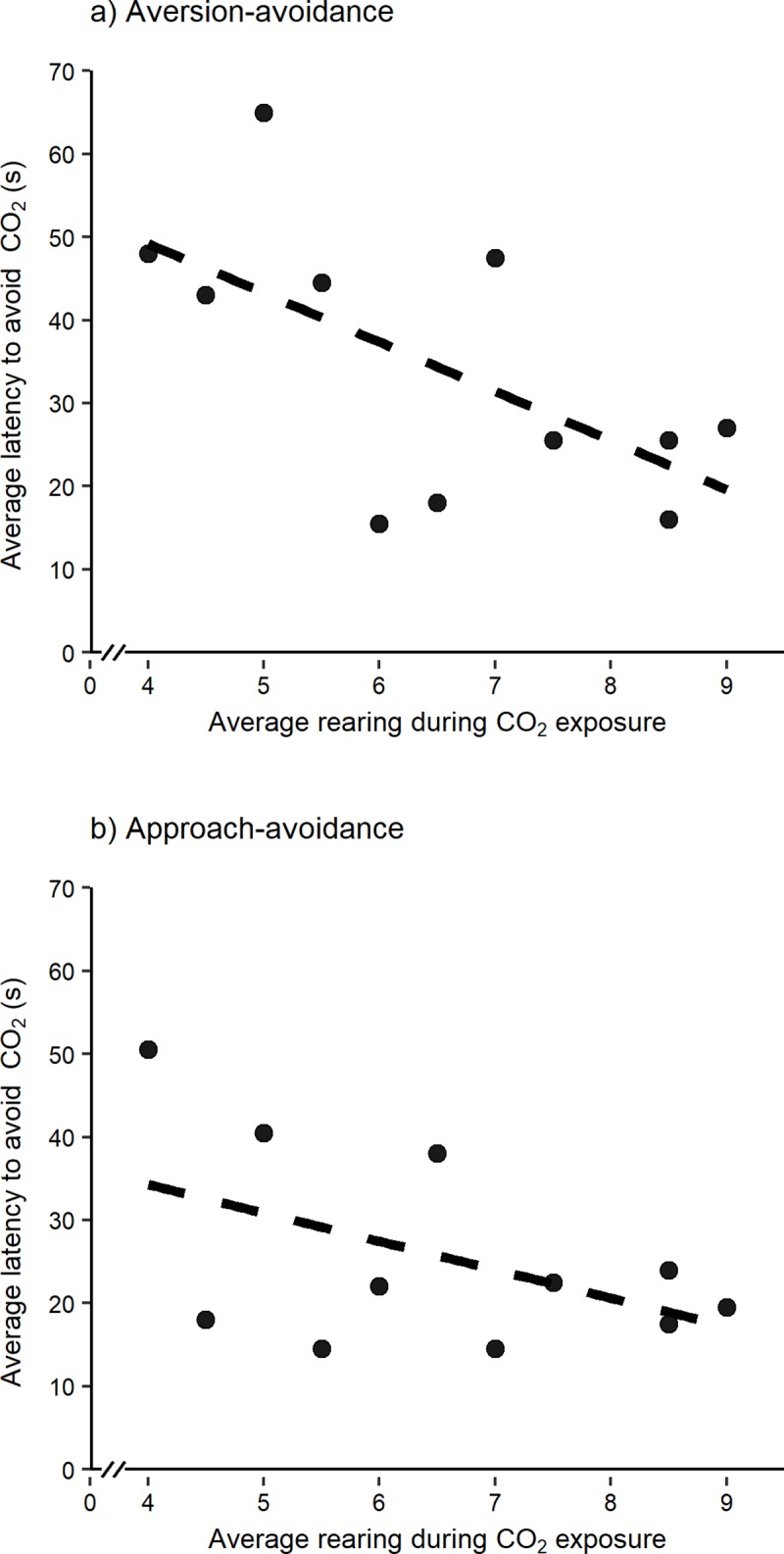
Forced exposure and strength of aversion. Relationship between the average frequency of rearing during forced exposure to CO_2_ and the average latency to avoid CO_2_ in the a) aversion-avoidance (n = 11 rats) and b) approach-avoidance tests (n = 11 rats).

Average rearing during forced exposure to CO_2_ was negatively correlated with latency to avoid CO_2_ in the aversion-avoidance test (r = -0.62, df = 9, p = 0.04; Fig 4A). There was less evidence of a negative relationship between rearing and latency to avoid CO_2_ in the approach-avoidance test (r = -0.49, df = 9, p = 0.13; Fig 4B).

## Discussion

### Active and passive responses during forced exposure

In agreement with another study using similar flow rates (~ 17 CO_2_ chamber vol. min^-1^) and air exposure as a control treatment [9], we found that the change from baseline in rearing and locomotion was higher with CO_2_ than the control. The change in locomotion (line-crossing) from baseline was approximately 3.5 and 3 times higher during CO_2_ exposure and re-exposure, respectively, compared to O_2_ treatment; this change from baseline was 3 times greater than during fox scent exposure and re-exposure. The change from baseline for rearing was approximately 2 and 5 times higher during CO_2_ exposure and re-exposure, respectively, compared to O_2_ treatment; this change from baseline was 4 and 5 times greater than during fox scent exposure and re-exposure, respectively.

We found no evidence of increased immobility during CO_2_ exposure, and the change from baseline in this measure was lower with CO_2_ than with O_2_ and fox scent treatments during re-exposure. The lack of increase in passive responses during CO_2_ exposure may be due to the strain used in this experiment. Winter et al. [25] reported that when exposed to 10% static CO_2_, Long Evans responded with higher immobility times than Wistar and Sprague Dawley rats. However, within the same strain variation between studies still exists. Sprague Dawley rats exposed to the CO_2_ challenge (rapidly increasing concentration stabilizing at 20% CO_2_ after 5 min), increased active but not passive responses in one study [39] but showed a decreased response in others [26,27]. Strain differences in responses to fox scent have also been reported. Sprague Dawley and Long Evans rats increased immobility when exposed to fox scent, and this response is greater than that of Wistar rats [40,41]. In the current study we found an increase in active responses in Sprague Dawley rats, but other authors using the same strain reported an absence of active responses to CO_2_ exposure [19,20]. We suggest that strain differences may be important but are unlikely to explain all of the between study differences in active and passive responses.

CO_2_ concentration and the possibility of avoiding exposure might also influence responses. The type and intensity of rat defensive behaviors expressed when confronting threating stimuli is flexible, sensitive to specific features of the stimuli, and situation-dependent. For example, the behavioral responses of rats vary in intensity depending on predator scent concentration [37], but if provided with the opportunity, rats will actively avoid the scent [42,43]. Passive responses were reported for rats exposed to 10% static CO_2_ [25]. Using the 20% CO_2_ challenge, Johnson et al. [26,27] report that rats froze when CO_2_ concentrations reached around 15%. For rats exposed to a medium flow rate of CO_2_, the peak of active responses occurred at around 20% CO_2_ [9]. Another study found that when rats were provided the opportunity to escape (in an approach-avoidance experiment) they tolerated 10% static CO_2_ for around 5 min and consumed all available food rewards, but at 15% CO_2_ rats remained 46 s and consumed only a few of the available food rewards [13]. Other studies have found that when exposed to medium flow rates, rats avoided an average of 18.4% CO_2_ [13]. It is plausible that at lower inescapable concentrations (between 10 to 15% CO_2_) CO_2_ elicits freezing, but if an escape route is provided rats will tolerate similar CO_2_ concentrations if motivated to do so. However, higher CO_2_ concentrations (over 18% CO_2_) appear to elicit active responses and are always avoided by rats.

### Within- and between-treatment consistency in active and passive responses

We found that rearing was consistent between the first and second forced exposures to CO_2_, but not between exposures to fox scent treatment. Rats increased rearing from baseline, indicating that rearing during test was an avoidance-motivated behavior (for a review, see [44]). In addition, previous work using similar flow rates has shown that during the first 20 s of gradual-fill, CO_2_ concentrations at the bottom of the cage tended to be 7% higher than at the top of the cage [9]. Hence, consistency in rearing responses between exposures suggests that rat motivation to avoid CO_2_ during forced exposure, rather than variation in their motivation to explore the cage.

In the current study, forced exposure to CO_2_ and fox scent failed to elicit consistent passive responses in rats. Fox scent consisted of the compound TMT which is found in fox feces [45]. Although it has been previously reported that rats respond to TMT with immobility (e.g. [37,46,47]), some studies have found a lack of a response (e.g. [48,49]). The different factors that could account for the absence of passive responses during forced exposure to fox scent have been reviewed by Fendt and Endres [40]. Since variation in rat coping strategies is characterized by active and passive responses [24], the lack of consistency in passive responses during CO_2_ exposure suggests that variation in rat responses during CO_2_ exposure do not represent general differences in how individuals cope with threatening stimuli; this conclusion is tempered by the lack of consistency in passive responses during the fox scent treatment.

### Consistency in the strength of aversion to CO_2_

In the current study, rats avoided on average 9 and 11% CO_2_ in the approach-avoidance test during exposure and re-exposure, respectively. These concentrations are lower than those reported in previous studies using similar flow rates. For example, using medium flow rates (between 15 and 20% CO_2_ cage volume min^-1^) rats avoided on average between 15 and 18% CO_2_ [13,15,17]. It is possible that tolerance to CO_2_ increases with exposure and experience in these tests. When rats were repeatedly exposed to CO_2_ medium flow rates in the approach-avoidance test, the average tolerance to CO_2_ increased from ~14% CO_2_ in the first three trials, to 18% CO_2_ the last exposure [16]. Humans habituate to CO_2,_ reducing chemoreceptor sensitivity [50] and anxiety [51], and increasing the threshold for the onset of air hunger (dyspnea) and respiratory response [52]. However, our results also differ from those obtained from naïve rats (~15% CO_2_) [16]. The rats tested in the current study had previous experience of forced CO_2_ exposure. This forced exposure may have affected their willingness to tolerate the gas in the later tests. It has been shown that acute (over 35% static CO_2_) exposure to CO_2_ produces conditioning which resists extinction in rats [53]. It is worth noting that in the current study during O_2_ training rats also left the bottom cage earlier than reported in previous studies where training was done with air (between 62 and 74 s earlier) [16,17]. We suggest that the high-oxygen environment created by O_2_ flow (as opposed to airflow) was aversive; rats are able to discriminate between different above atmospheric concentrations of O_2_ [54].

Individual variability in strength of aversion to CO_2_ may indicate variation in CO_2_ sensitivity. Previous studies using approach-avoidance testing have reported between individual variability in CO_2_ aversion [15,17]. We found that rat identity was an important source of variation in CO_2_ thresholds of aversion for the aversion- and the approach-avoidance tests. Within each aversion test, latency to avoid CO_2_ was consistent between two exposures, and active defense responses during CO_2_ forced exposure were associated with latency to avoid CO_2_ in the aversion tests.

In the current study we found no evidence of consistency between aversion assessed through approach-avoidance and aversion-avoidance. These results indicate that aside from CO_2_ sensitivity, other factors may influence variation in rat aversion to CO_2_. There are a number of situational-elicited individual differences that might account for this variation. In both aversion tests, it was assumed that all rats were strongly motivated to approach or avoid the paired stimuli (sweet rewards and a brightly lit chamber, respectively) used to assess the strength of aversion to CO_2_. Food deprived rats are motivated to avoid light exposure (1650 lux) even at the cost of losing a food reward [55]. In addition, even without food deprivation, rats are highly motivated to approach sweet rewards [15]. However, individual rats vary in their motivation to approach and avoid these paired stimuli. Rats consistently vary in light aversion [56] and in their motivation to work for sucrose [57–59]. Between-subject variation in aversion- and approach-avoidance tests is likely influenced by motivational differences in addition to CO_2_ sensitivity.

### Responses to forced exposure and strength of aversion to CO_2_

In the current study we found that rearing during CO_2_ forced exposure was negatively correlated to the latency to avoid CO_2_ in the aversion-avoidance test. Consistency in rat responses to CO_2_ within testing situations, and between forced exposure and aversion-avoidance tests, provide evidence of rat variation in CO_2_ sensitivity. In humans, individuals differ in the type and intensity of the responses when inhaling CO_2_ [29,60]. For example, anxiety was experienced by 60% of healthy humans during prolonged inhalation of low CO_2_ concentrations (7% CO_2_ during 20 min), and this experience was consistent between exposures [34]. Feelings of immobility and desire to flee were experienced by 13% and 20% of healthy individuals, respectively, during shorter exposure to medium CO_2_ concentrations (20 s exposure to 20% CO_2_) [61]. Panic attacks are experienced by healthy individuals following a double inhalation of 35% CO_2_ [62], but panic attacks and anxiety are consistently elicited with a single inhalation of 35% CO_2_ in panic disorder patients [35].

### Study limitations

The failure to detect some relationships in the current study could be due to small sample size [38]. Given that little previous work has addressed the issues considered in this paper we suggest that the current results are of value, although with any study there is merit in constructive replication of these tests by other laboratories. In addition, this study included multiple tests, potentially increasing the likelihood of type I error (see [63]). Our approach to reducing this risk was to focus on (and provide statistical tests for) only a few relationships for which we had strong predictions. An alternative approach would be to omit inferential analyses and report correlation coefficients descriptively varying from negligible to very high (e.g. [64]).

## Conclusions

Rats varied consistently in their responsiveness to CO_2_ exposure. If these responses relate to the animal’s affective states, then the emotional experience when killed with CO_2_ may also vary among rats. These results reinforce the importance of assessing affective states at the level of the individual, rather than relying on measurements of central tendency (discussed by [65]). In addition, accounting for individual differences may allow for of a better understanding experimental results, perhaps especially when assessing animal welfare [66]. Overall, our results indicate that variation in rat responses to CO_2_ exposure is situation-specific and relate to variation in CO_2_ sensitivity. CO_2_ concentrations well below those necessary to induce unconsciousness were aversive to all rats, indicating that CO_2_ exposure compromises rat welfare even for the least sensitive rats.

## Supporting information

S1 Appendix Bleach treatment. Experimental design and testing procedure of the bleach exposure study.
(DOCX)

S2 Appendix Study timeline. Timeline of the three experiments performed in the current study.
(DOCX)

S1 Dataset Forced exposure. Data obtained from Experiment 1: forced exposure.
(XLSX)

S2 Dataset Aversion- and approach-avoidance. Data obtained from Experiments 2 and 3: aversion- and approach-avoidance, respectively.
(XLSX)

S3 Dataset Bleach treatment. Data obtained from the bleach exposure study.
(XLSX)
