# Supplementary material for: Evidence for consistent individual differences in rat sensitivity to carbon dioxide
Source: PLoS One. 2019 Apr 24;14(4):e0215808. doi: 10.1371/journal.pone.0215808 (PMC6481838; doi:10.1371/journal.pone.0215808)
Supplement: S1 Appendix — Experimental design and testing procedure of the bleach exposure study. (DOCX) [file pone.0215808.s001.docx]

**S1 Appendix. Bleach treatment.** Experimental design and testing procedure of the bleach exposure study

*Experimental design*

All rats tested in Experiment 1 were forced exposed to bleach (2 ml; The Clorox Company, [CA, USA](https://www.google.ca/search?safe=active&hl=en&authuser=0&biw=1366&bih=643&q=Oakland+California&stick=H4sIAAAAAAAAAOPgE-LUz9U3MM3NLTFQ4gAxU5ItTbW0spOt9POL0hPzMqsSSzLz81A4VhmpiSmFpYlFJalFxQC6o8gXQwAAAA&sa=X&ved=0ahUKEwiAt_Oi74LLAhUE-mMKHU4wAKAQmxMInwEoATAa). Order of exposure was allocated using three 4x4 Latin squares (considering the three treatments of the main experiment, four rats and four treatments per Latin square). Three days later, the same rats were re-exposed to bleach, allocating treatment order again in three 4x4 Latin squares. Each rat was tested only once a day. Tests were performed between 900 h and 1700 h, and each rat was tested at similar hours within and across all experiments.

*Testing procedure*

Rats were individually placed in the experimental cage covered with the baseline lid, and remained in it for 5 min (baseline). The lid was then replaced with the experimental lid. The tea ball attached to the experimental lid contained a cotton ball soaked with 2 ml of bleach. Tests lasted 15 min.
