# Supplementary material for: Evidence for consistent individual differences in rat sensitivity to carbon dioxide
Source: PLoS One. 2019 Apr 24;14(4):e0215808. doi: 10.1371/journal.pone.0215808 (PMC6481838; doi:10.1371/journal.pone.0215808)

**S2 Appendix. Study timeline.** Timeline of the three experiments performed in the current study

All rats were tested in each of the three experiments (i.e. Forced exposure, Aversion-avoidance and Approach-avoidance) in the same order and timeline (presented below)


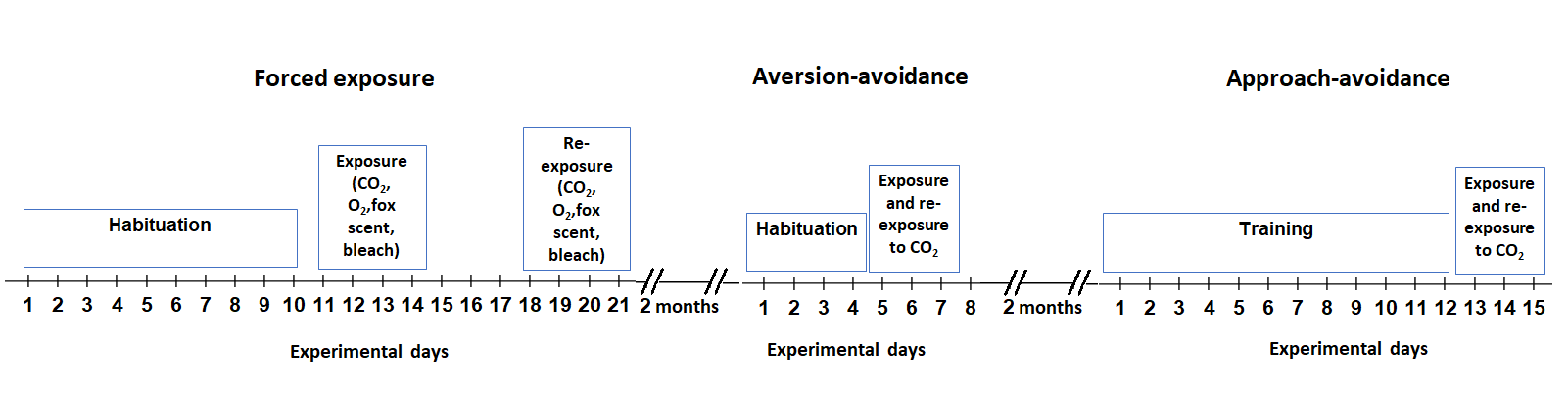

Supplement: S2 Appendix — Timeline of the three experiments performed in the current study. (DOCX) [file pone.0215808.s002.docx]
